# Cryoballoon ablation guided by a novel wide-band dielectric imaging system

**DOI:** 10.3389/fcvm.2022.967341

**Published:** 2022-08-09

**Authors:** Laura Rottner, Julius Obergassel, Ilaria My, Paulus Kirchhof, Feifan Ouyang, Bruno Reissmann, Andreas Metzner, Andreas Rillig

**Affiliations:** ^1^Department of Cardiology, University Heart and Vascular Center Hamburg, Hamburg, Germany; ^2^Institute of Cardiovascular Sciences, University of Birmingham, Birmingham, United Kingdom

**Keywords:** cryoballoon ablation, occlusion tool software, dielectric imaging system, pulmonary vein ablation/isolation, high resolution imaging

## Abstract

**Background and aim:**

To investigate the feasibility, acute efficacy, periprocedural safety, and procedural parameters of CB-based PVI guided by KODEX-EPD using different occlusion tool software versions.

**Methods and results:**

A total of 173 patients (60/173 (35%) paroxysmal AF, 64 ± 12 years, 66/173 (38%) female), underwent CB-based PVI guided by KODEX-EPD between August 2019 and October 2021. Acute PVI was achieved in all the patients. Total fluoroscopy time and dye volume were 13.9 [10.6–19.4] min and 47.5 [20–70] ml. Periprocedural complications occurred in 3 (2%) of the 173 patients. As part of the continued development program, different software versions were used, including 1.4.6 on 38 (22%), 1.4.6a on 33 (19%), 1.4.7 on 41 (24%), and 1.4.8 on 61 (35%) of the patients. Outcomes were compared between software versions by a univariate analysis. Sensitivity analyses were performed to account for confounders. Median fluoroscopy time was decreased by 6.4 min (37.3%), and the median volume of dye was decreased by 32.5 ml (52%) from versions 1.4.6 to 1.4.8. Software version was a significant predictor of fluoroscopy time and dye volume, while reductions in procedure duration and dose area product were observed but mainly explained *via* confounders.

**Conclusion:**

CB-based PVI guided by KODEX-EPD is feasible and safe. Progressive software improvements appear to be associated with lower fluoroscopy duration and dye use. Further studies are needed to evaluate the advantage of KODEX-EPD-guided compared to conventional CB-PVI.

## Introduction

Pulmonary vein isolation (PVI) is gold standard in interventional rhythm control for patients suffering from symptomatic atrial fibrillation [AF, (1)]. Permanent transmural lesion formation remains decisive for long-term clinical outcomes after PVI (2). During cryoballoon (CB)-based PVI, complete pulmonary vein (PV) occlusion with an inflated balloon is pursued to achieve permanent isolation of the veins since persistent blood flow will prevent adequate lesion formation. CB occlusion assessment by fluoroscopy and dye-injection is widely conducted during CB-PVI procedures, requiring additional ionizing radiation and use of a contrast dye (3, 4).

KODEX-EPD (EPD Solutions, Philips, Netherlands) is a cardiac imaging system that can be used to guide CB-based PVI (5). With its ability to assess dielectric properties of structures around an inserted catheter, the system can assess occlusion of PVs (6–8). Improved software versions with improved occlusion workflows aiming for faster and more accurate PV occlusion assessment were introduced recently.

Here, we report the feasibility, acute efficacy, and periprocedural safety of KODEX-EPD and its occlusion tool. We also report the effect of different software versions on procedural parameters during CB-based PVI.

## Methods

### Study population

Consecutive patients undergoing CB-based PVI guided by 3D wide-band dielectric imaging between August 2019 and October 2021 were retrospectively analyzed. The analysis was approved by the local ethics committee (WF-021/20). Data analysis and handling were performed in accordance with the Declaration of Helsinki.

### Procedural management

Prior to the procedure, transthoracic and transesophageal echocardiography was performed to rule out intracardiac thrombi and to assess LA diameter. No additional pre-procedural imaging was performed. AF ablation was performed on uninterrupted oral vitamin K anticoagulation with an INR of 2–3 on the day of the procedure. In patients treated with directoral anticoagulants (DOACs), anticoagulation was stopped the evening before the procedure.

Our ablation strategy for CB-based PVI in conjunction with KODEX-EPD was previously described in detail (9, 10). In brief, a 3D anatomical image of the LA and the PVs was created in a standardized sequence using a 20-mm circular mapping catheter (Achieve; Medtronic Inc., Minneapolis, United States) and a steerable sheath prior to ablation. After acquisition of the LA and PV shell, the system renders an additional PANO-view, a world-map like endocardial view of the LA with its specific structures such as the PVs, mitral valve, and left atrial appendage. Because of our institutional standard, selective PV angiography was additionally performed in order to identify the individual PV anatomy in 53% of the patients (92/173). During the course of the study, selective PV angiographies were waived and instead KODEX-EPD-only imaging was conducted to assess LA and PV anatomy in the other 43% of the patients (81/173). In all the patients, the fourth-generation CB was used in conjunction with a time-to-effect guided ablation strategy. Verification of complete PV occlusion, assessed with the KODEX-EPD's occlusion tool and confirmed by fluoroscopy, was required prior to the start of ablation. After documentation of PVI, the freeze cycle was prolonged for additional 120 s. If no real-time PVI signal recordings could be obtained, a standard freeze cycle of 180 s was applied. No empiric bonus freeze cycles were applied.

Pericardial effusion was ruled out in all the patients by transthoracic echocardiography after the procedure. Low molecular-weight heparin was administered in patients on vitamin K antagonist when the International Normalized Ratio (INR) was below 2 until a therapeutic INR of 2 to 3 was reached again. Pre-existing therapy with NOACs was resumed 6 h after the procedure.

### Different software versions and improved workflows of KODEX-EPD and its occlusion tool

**Figure 1:**
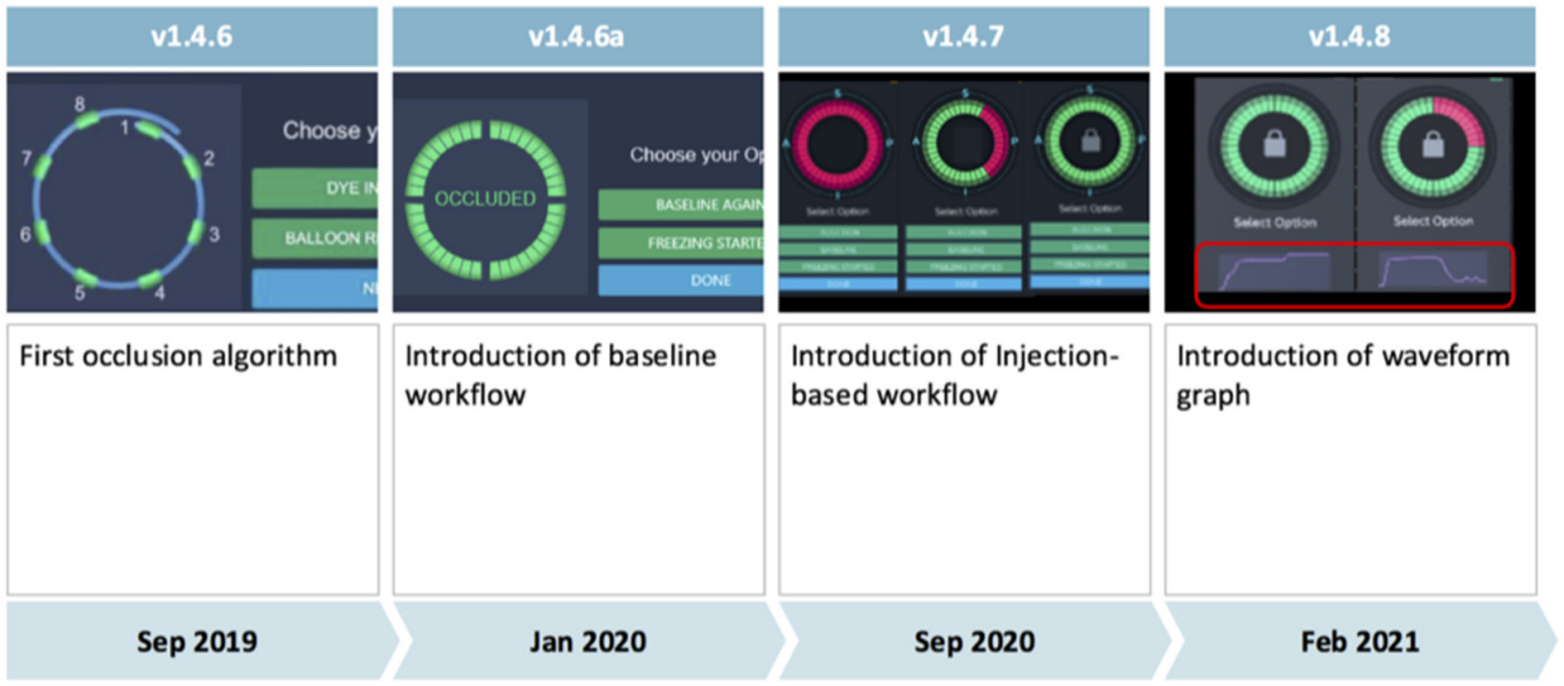
Evolution of the KODEX-EPD occlusion tool from software version 1.4.6 to software version 1.4.8. The injection-based workflow assesses injected contrast dye or saline along the acquiring circular mapping catheter and was used in all patients since release of software version 1.4.7. It superseded the previous baseline workflow. Further improvements are discussed in the main manuscript's methods section.

Four software versions (1.4.6, 1.4.6a, 1.4.7, and 1.4.8) were used during the study (Figure 1).

Major differences between the occlusion tool in its initial version 1.4.6 and the following version (1.4.6a) include an improvement of the algorithm for occlusion detection based on clinical data collected with software version 1.4.6, as well as employment of machine learning algorithms, and the ability to mark a shadow position of the circular mapping catheter to provide guidance for its optimal placement during occlusion assessment.

**Figure 2:**
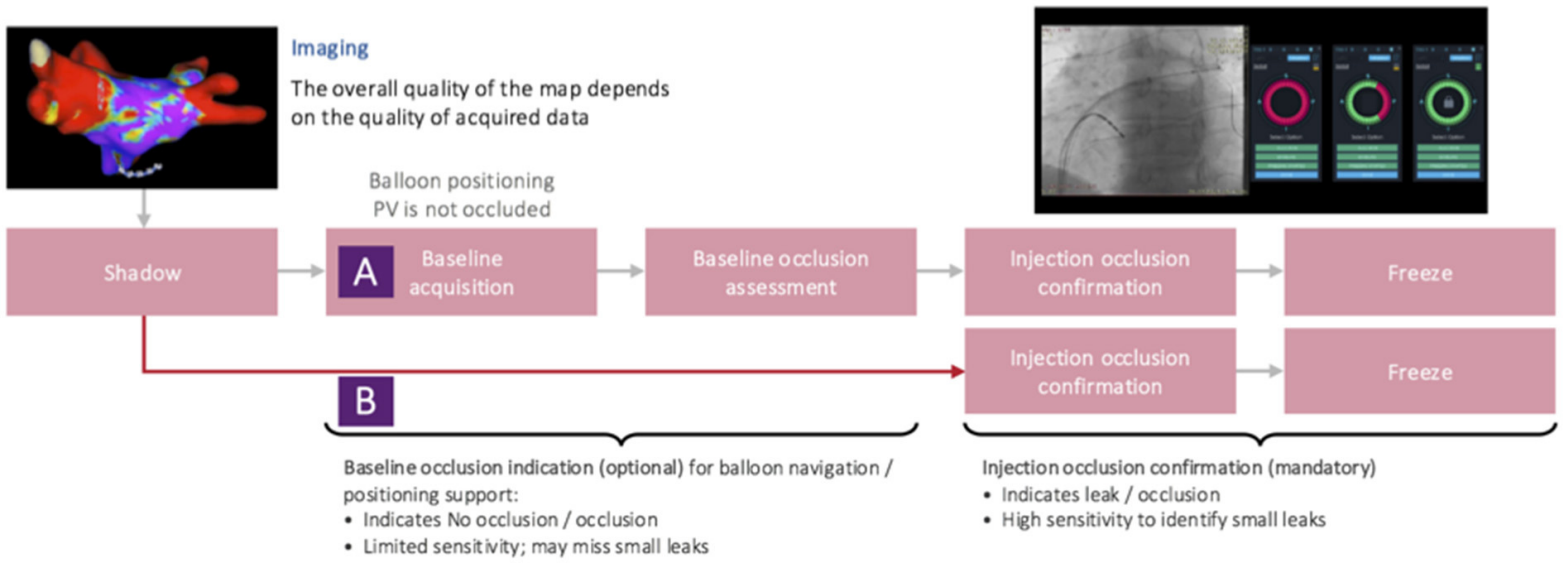
Baselining and injection-based workflow steps of KODEX-EPD's occlusion tool.

Initially (in software versions 1.4.6 and 1.4.6a), the occlusion tool required baselining, which has been reported to be feasible with the potential to accurately assess PV occlusion (6, 7). However, it added another step to the conventional cryoablation workflow. Aiming for faster and more accurate assessment of PV occlusion *via* KODEX-EPD, the injection-based workflow for the occlusion tool has been lately added in version 1.4.7. Furthermore, in 1.4.7, multi-map-display was introduced that, inter alia, enabled tagging of the phrenic nerve within the right atrium (RA) in parallel to LA mapping. With the introduction of software version 1.4.7, the injection-based occlusion workflow was used on all the patients (Figure 2).

With software version 1.4.8, the waveform graph for occlusion monitoring in parallel to occlusion status indication was implemented. Furthermore, 1.4.8 enabled faster imaging, better catheter localization and motion, and respiration compensation. In addition, there was improvement in catheter stability and imaging during pacing with 1.4.8.

### Data handling and statistical analyses

Data mining of procedural records identified relevant ablation cases and extracted procedural data. Missing data points and extreme outliers (outside of a range of three standard deviations around the mean) were manually double-checked. If variables were not available after manual assessment, the procedure was included with the available variables. Procedural duration, fluoroscopy duration, and dose area product could be transformed into normally distributed variables. Dye use was considered left-censored and was not transformed. Procedure date and information whether PV angiography was performed were considered as possible confounders (cf. Supplementary material).

Normally transformed outcome parameters were analyzed by analysis of variance (ANOVA). A Tobit regression model was fitted to assess the effect of software version on used contrast as a left-censored outcome variable. An additional sensitivity analysis was performed to evaluate the impact of the two possible confounders PV-angiography and procedure date *via* generalized linear models, by analysis of covariance (ANCOVA), and with additional Tobit models (cf. Supplementary material).

All data handling, harmonization, and statistics were performed in Python (v 3.9.9) using common data science packages (e.g., Pandas, NumPy, SciPy, Bioinfokit, and Pingouin) and R (v 4.1.3). Figures were calculated in Python using the plotly library and post-processed in Adobe Illustrator (v 26).

## Results

### Patient population

A total of 173 consecutive patients suffering from symptomatic AF and who underwent CB-based PVI in conjunction with KODEX-EPD were analyzed. Sixty (35%) of the 173 patients suffered from paroxysmal AF. Median patient age was 65 years [IQR 58; 74 years]. Sixty-six (38%) of them were female. Patient characteristics are summarized in Table 1.

**Table 1:** Baseline patient characteristics.

		**Overall**	**1.4.6**	**1.4.6a**	**1.4.7**	**1.4.8**
N		**173**	**38**	**33**	**41**	**61**
Age (years)		64.8 [57.8, 73.5]	67.5 [59.5, 75.9]	64.5 [60.4, 72.9]	63.4 [56.3, 70.7]	63.2 [55.7, 75.0]
Sex, *n* (%)	Male	107 (61.8)	22 (57.9)	27 (81.8)	24 (58.5)	34 (55.7)
AF type, *n* (%)	Non-PAF	113 (65.3)	24 (63.2)	25 (75.8)	28 (68.3)	36 (59.0)
	PAF	60 (34.7)	14 (36.8)	8 (24.2)	13 (31.7)	25 (41.0)

Continuous data are reported as medians (25th and 75th percentiles). Categorical data are presented as n (%).

AF, atrial fibrillation; PAF, paroxysmal atrial fibrillation.

### Overall procedural parameters

Total procedure and fluoroscopy times were 81 [67, 98] and 13.9 [10.6–19.4] min, respectively. Overall dose area product and volume of dye were 501 [281.9, 844.6] cGy × cm^2^ and 47.5 [20–70] ml.

### Univariate analysis of procedural parameters

**Figure 3:**
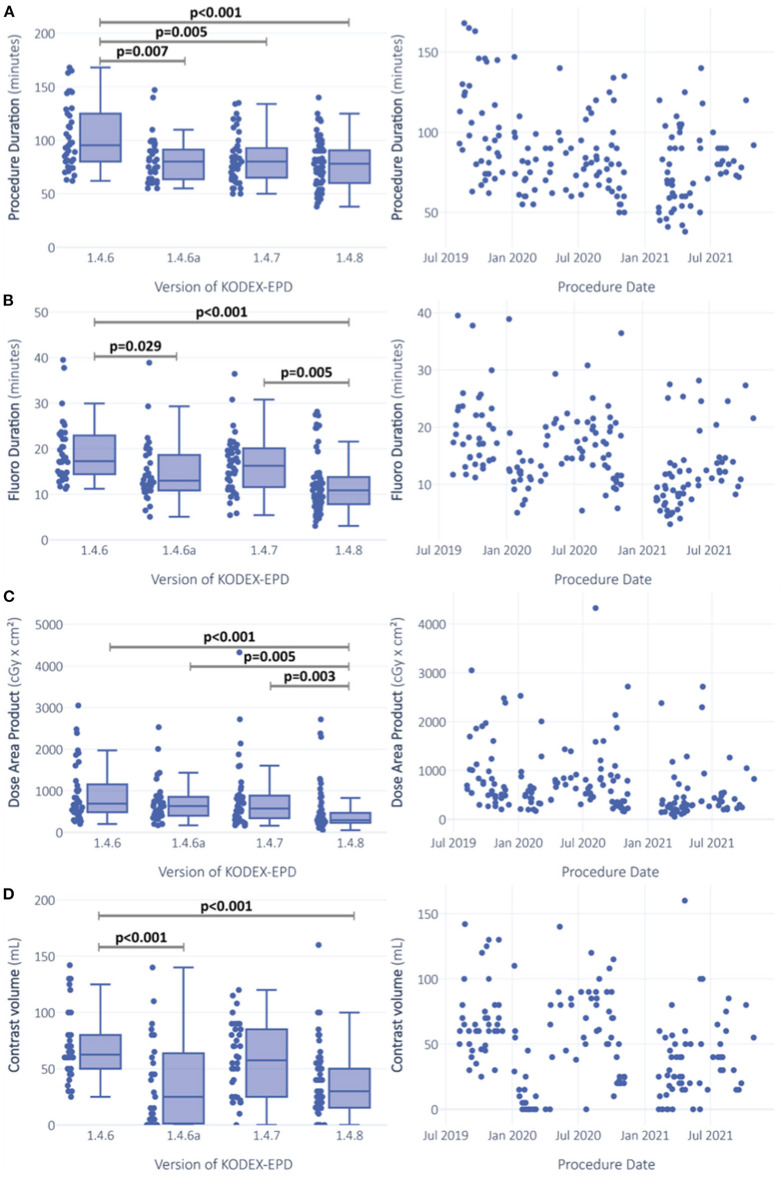
Comparison of procedural outcomes between software versions and over time. Procedure duration **(A)**, fluoroscopy duration **(B)** and dose area product **(C)** were gaussian-transformed and analysis was performed via ANOVA. ANCOVA sensitivity analysis was additionally performed with adjustment for procedure date and whether PV-angiography was performed or not. Left censored variable usage of contrast dye **(D)** was analyzed via Tobit-regression. Significance is indicated for ANOVA *post-hoc* testing with Turkey-correction for procedure duration **(A)**, fluoroscopy duration **(B)** and dose area product **(C)**. For usage of contrast dye **(D)**, significant predictors in the unadjusted Tobit-model are indicated.

Software version showed significant effects on procedure duration, fluoroscopy duration, and dose area product in the initial ANOVA testing (Figures 3A–C). Most version differences in *post hoc* testing were observed for fluoroscopy duration (Figure 3B). The unadjusted Tobit regression revealed significant effects of software versions 1.4.6a and 1.4.8 (Figure 3D, Supplementary Table 1).

#### Software-version-driven reductions of fluoroscopy duration and used volume of contrast die

Software versions affected fluoroscopy duration and dye usage (Figures 3B,D). Median fluoroscopy duration in procedures performed with version 1.4.6 was 17.3 [IQR 14.4; 22.7] min, and it was reduced by 6.4 min (37.1%) with version 1.4.8 (median 10.9 [IQR 7.9, 13.8] min). After adjustment for the abovementioned confounders, software version remained the strongest predictor for fluoroscopy duration with the highest effect size (*p* < 0.001, $\eta_{\text{p}}^{2}$ = 0.113) among all the tested predictors (PV angiography: *p* < 0.001, $\eta_{\text{p}}^{2}$ = 0.066; procedure date: *p* < 0.001, $\eta_{\text{p}}^{2}$ = 0.079; Table 2, Supplementary Figure 1).

**Table 2:** Results of analysis-of-variance (ANOVA) testing and ANCOVA adjusting for procedure date and PV angiography.

	**Unadjusted ANOVA**		**Adjusted ANCOVA**	
	**p**	**η^2^**	**p**	** $\eta_{\text{P}}^{2}$ **
**Procedure duration**				
- **Software version**	<0.001	0.124	0.111	0.035
- procedure date			0.003	0.004
- PV angiography			0.061	0.063
**Fluoroscopy duration**				
- **Software version**	<0.001	0.199	<0.01	0.113
- Procedure date			<0.01	0.079
- PV angiography			<0.01	0.066
**Dose area product**				
- **Software version**	<0.001	0.148	0.302	0.022
- Procedure Date			0.395	0.004
- PV angiography			0.004	0.049

Reported are unadjusted and adjusted p-values and effect size measures (eta square η^2^ and partial eta square $\eta_{\text{P}}^{2}$) for software version for the outcome parameters procedure duration, fluoroscopy duration, and dose area product.

**Figure 4:**
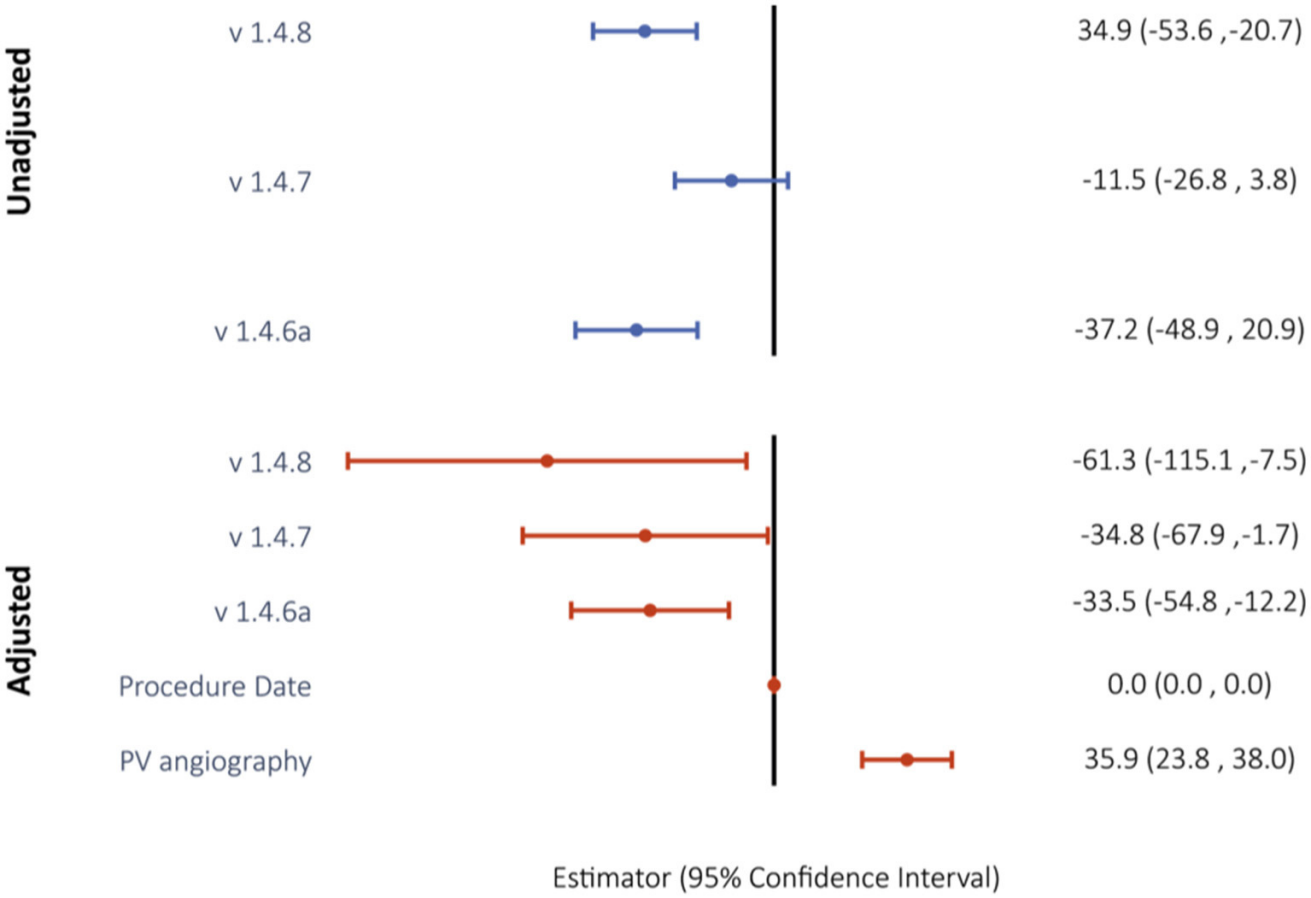
Unadjusted and confounder-adjusted Tobit regression model to evaluate the impact of software version on use of contrast dye. Reported are Tobit estimates with 95% confidence intervals in brackets. Version 1.4.6 was used as the model constant.

Median volume of dye was decreased with versions 1.4.6 to 1.4.8 by 32.5 ml (52%) from 62.5 to 30 ml. The impact of predictors on the left-censored variable volume of dye was analyzed *via* Tobit regression models. With software version 1.4.6 as the constant, software versions 1.4.6a and 1.4.8 were predictors with negative estimates for contrast dye (both *p* < 0.0001; Figure 3D, Supplementary Table 1). In the adjusted multivariable regression, all the software versions were independent predictors of contrast volume with negative estimates (Figure 4).

#### Procedure duration and dose area product

Median procedure duration was 95.5 [IQR 80; 124.5] min with version 1.4.6 and was reduced to 78 [IQR 60; 90] min with version 1.4.8, which is a median reduction of 17.5 min (18.3%) (Figure 1A). In adjusted regression models, changes in procedure duration seemed to be mainly explained by the performed PV angiography (*p* < 0.001, $\eta_{\text{p}}^{2}$ = 0.063), but the effects of software version were still significant (*p* = 0.033, $\eta_{\text{p}}^{2}$ = 0.035) (Table 2). There was a highly significant effect of PV angiography in the *post hoc* testing for this model (*p* < 0.001), while the *post hoc* results of software version were not significant anymore (Table 2).

Similar observations were made for dose area product, which was reduced from 692.8 [486.8, 1126.4] cGy × cm^2^ with 1.4.6 to 293.7 [233.4, 462] cGy × cm^2^, a median reduction of 399.1 cGy × cm^2^ (57.6%). While the initial ANOVA was significant (*p* < 0.0001) with *post hoc* differences between versions 1.4.6 and 1.4.8 (*p* < 0.0001), 1.4.6a and 1.4.8 (*p* = 0.004), and 1.4.7 and 1.4.8 (*p* = 0.003) (Figure 3C), in the adjusted ANCOVA, the effect seemed to be mainly explained by the PV angiography variable (*p* = 0.004, $\eta_{\text{p}}^{2}$ = 0.049), and software version was not significant anymore after the adjustment (*p* = 0.302).

### Feasibility and acute efficacy

All targeted PVs were successfully isolated and either verified by TTI or absence of recording of PV potentials after ablation.

### Periprocedural safety

One case of asymptomatic phrenic nerve palsy not resolving until discharge was observed. Furthermore, one case of procedure-related transient ischemic attack (TIA) was documented. In another patient, mild post-interventional pericarditis occurred. No further acute complications were observed.

## Discussion

This is the first study reporting on a large series of CB-based PVI procedures guided by the novel wide-band dielectric imaging system KODEX EPD (EPD-solutions; Philips, Netherlands). This study aimed to report on the feasibility, acute efficacy, and periprocedural safety of different software versions of the occlusion tool on procedural parameters during KODEX-EPD guided CB-PVI.

The main findings of the analysis are:

- CB-based PVI in combination with KODEX-EPD and the occlusion tool is feasible and safe.
- CB-based AF ablation in conjunction with KODEX-EPD and its occlusion tool is associated with high acute ablation success.
- Iterative software updates were associated with shorter fluoroscopy times and reduced dye volumes to achieve successful CB-based PVI.

To our knowledge, this study reports on the largest series of CB-based PVI procedures guided by the novel wide-band dielectric imaging system KODEX EPD (EPD-solutions; Philips, Netherlands).

### Feasibility, safety, and acute efficacy of CB-based PVI in conjunction with wide-band dielectric imaging

In this study, all targeted PVs were isolated successfully. The rate of minor complications (one asymptomatic phrenic nerve palsy, one TIA) is in line with expected and published complication rates. Hence, our data confirm that CB-based PVI in conjunction with KODEX-EPD is feasible and safe and associated with high rate of acute ablation efficacy. Further analysis on long-term arrhythmia-free survival is desirable.

Whereas in principle any diagnostic catheter can be used for LA imaging, it appears reasonable to use the circular mapping catheter (Achieve; Medtronic, Inc.) during a CB ablation procedure. The Achieve catheter can be fully visualized with the KODEX-EPD system. The “PANO view” enables the operator to easily navigate the mapping catheter in the LA without a relevant learning curve. This PANO view might also help to avoid unintentional positioning of the circular catheter into the LV and therefore might additionally prevent severe complications such as catheter entrapment in the mitral valve (10, 11).

### Potential benefits of KODEX-EPD during CB-based AF ablation procedures

The potential value of implementing an imaging system such as KODEX-EPD during CB ablation procedures is of several aspects. In particular, benefits may be obtained by a better understanding of the anatomy, potential optimization of procedural steps, and reduction of fluoroscopy exposure and dye.

Although several institutions use pre-procedural imaging techniques such as computer tomography and magnetic resonance imaging prior to ablation procedures, both imaging modalities have certain disadvantages such as fluoroscopy exposure, need for application of contrast dye (12, 13), and limitations in patients with implanted cardiac devices. Besides that, pre-procedural imaging is only of limited value during the CB procedure itself, as image integration in a 3D mapping system would offer the opportunity for intraprocedural real-time guidance of the diagnostic catheter in the LA and PV branches, which is recommended to sufficiently position the CB. For visualization of PVs, selective angiographies are still considered as the method of choice by most operators while being associated with the need for additional fluoroscopy and dye injection (3). The latter is associated with allergic reactions or acute and chronic kidney damage (14).

Fluoroscopy-guided CB-based PVI requires longer fluoroscopy times than radiofrequency (RF) based PVI (3). Although some advances were made aiming for fluoroscopy reduction (15), radiation exposure remains a major limitation of CB procedures (3, 16). With the novel KODEX-EPD imaging system, a 3D shell of the LA can be rapidly and accurately created prior to ablation (17). Furthermore, the system was proven to accurately visualize PVs and PV ostial diameters (10, 17). A particular strength of KODEX-EPD is that it allows to visualize cardiac structures without even reaching or touching them because of dielectric sensing of tissues surrounding the catheter tips (5).

The novel KODEX-EPD occlusion feature has been previously investigated in several smaller studies, and the results show that PV occlusion, as assessed with the KODEX-EPD occlusion tool, was adequate compared to the current “gold standard”, which is fluoroscopy and dye injection (7, 9). This study confirms the safety of the system and the effectiveness regarding acute PVI in a large series of consecutive procedures. In addition, our results suggest that consecutive improvements of software versions lead to lower fluoroscopy durations and dye usage during CB-based PVI. Largest effects of software version in the *post hoc* testing were observed for the outcome fluoroscopy duration.

Schillaci et al. (18) evaluated the impact of KODEX-EPD and the occlusion tool on procedural parameters, especially radiation and dye exposure, in a retrospective, single center study that enrolled 34 consecutive patients with paroxysmal AF. Here, 17 patients underwent a fluoroscopy-guided procedure while 17 patients underwent CB-PVI in combination with KODEX–EPD. They found that overall procedure time was comparable between the two groups, and that fluoroscopy time and dye use were significantly lower in the KODEX–EPD group. At 12-month follow-up, no differences between the study groups regarding atrial arrhythmia recurrence were observed (18). Our data further underlines potential reductions in fluoroscopy time and dye usage in KODEX-EPD-guided CB-PVI.

### Strengths and limitations

Strengths of the study include the analysis of consecutive patients in a standardized procedural workflow without further exclusion criteria. To our knowledge, this study represents the largest dataset of patients undergoing KODEX-EPD guided CB-based PVI.

Limitations include its retrospective design and non-randomized comparison of software versions. Besides, we only present data from a single center. The non-randomized comparison of software versions is a limitation but difficult to avoid during the innovation phase of the investigated system. Because the software versions were introduced sequentially, time (procedure date) was identified to be the most relevant confounder (e.g., due to learning curve using the system). However, the effects of software version on the mentioned procedural parameters remained significant, although slightly attenuated, after adjusting for procedure date. Based on the results of our study, we assume an overall independent effect of software version on fluoroscopy duration and dye usage.

Due to non-routine outpatient follow-up of patients after PVI in our clinical workflow, a detailed clinical follow-up on mid- and long-term effectiveness and safety of the reported procedures cannot be provided within this dataset. In this study, we focused on acute feasibility, safety, and efficacy, as well as procedural outcomes.

## Conclusion

CB-based PVI guided with KODEX-EPD and its occlusion tool is feasible, safe, and associated with high acute efficacy. The present data demonstrate the potential benefits of the novel wide-band dielectric imaging and mapping system to fluoroscopy- and dye-reduced CB-based AF ablation.

## Data availability statement

The raw data supporting the conclusions of this article will be made available by the authors, without undue reservation.

## Ethics statement

The studies involving human participants were reviewed and approved by Ethikkommission Hamburg. Written informed consent for participation was not required for this study in accordance with the national legislation and the institutional requirements.

## Author contributions

All authors listed have made a substantial, direct, and intellectual contribution to the work and approved it for publication.

## Conflict of interest

LR received travel grants and speaker's honoraria from EPD Solutions/Philips (KODEX-EPD). AR received travel grants from Biosense, Medtronic, St. Jude Medical, Cardiofocus, EP Solutions, Ablamap, and EPD Solutions/Philips (KODEX-EPD), and lecture and consultant fees from St. Jude Medical, Medtronic, Biosense, Cardiofocus, Novartis, and Boehringer Ingelheim. AM received speaker's honoraria and travel grants from Medtronic, Biosense Webster, Bayer, Boehringer Ingelheim, EPD Solutions/Philips (KODEX-EPD), and Cardiofocus. BR received speaker's honoraria and travel grants from Medtronic. PK received research support for basic, translational, and clinical research projects from the European Union, British Heart Foundation, Leducq Foundation, Medical Research Council (UK), and German Centre for Cardiovascular Research, from several drug and device companies active in AF, and has received honoraria from several such companies in the past but not in the last 3 years. PK is listed as inventor on two patents held by University of Birmingham (Atrial Fibrillation Therapy WO 2015140571, Markers for Atrial Fibrillation WO 2016012783).

The remaining authors declare that the research was conducted in the absence of any commercial or financial relationships that could be construed as a potential conflict of interest.

## Publisher's note

All claims expressed in this article are solely those of the authors and do not necessarily represent those of their affiliated organizations, or those of the publisher, the editors and the reviewers. Any product that may be evaluated in this article, or claim that may be made by its manufacturer, is not guaranteed or endorsed by the publisher.
