# Supplementary material for: Cryoballoon ablation guided by a novel wide-band dielectric imaging system
Source: Front Cardiovasc Med. 2022 Aug 9;9:967341. doi: 10.3389/fcvm.2022.967341 (PMC9395685; doi:10.3389/fcvm.2022.967341)

# Supplement

## **Supplement Methods**

Data mining was applied to identify all KODEX-EPD guided first-do CB-ablation procedures. All retrieved procedures were included in the analysis after manual review of correct classification. KODEX-EPD software version was attributed to each procedure according to procedure date and software installation dates.

For all procedures, the outcome parameters procedure duration, fluoroscopy duration, dose area product and used volume of contrast die were retrieved. Additional relevant input parameters retrieved besides software version were subtype of AF, patient age, patient sex, procedure date and performed PV angiography. Missing data points and extreme outliers (outside of a range of three standard deviations around the mean) were manually double checked at the original sources (original reports, handwritten periprocedural documents, fluoroscopy system). If variables were not available after manual assessment, the procedure was included with the available variables.

All outcome variables were tested for normal distribution using Shapiro-Wilk testing and evaluation of Q-Q-plots. Procedural duration, fluoroscopy duration and dose area product could be transformed in normally distributed variables via logarithmic- and 1/x-transformation for statistical analysis in general linear models (GLM). Used volume of contrast die was considered left censored and was not transformed, as multiple procedure aimed to minimize or dispense use of contrast die.

### Methodological identification of possible confounders

Procedures were clustered by installed software version as a surrogate for improvements to the occlusion tool at the time of the procedure. Since software versions were introduced sequentially, time of procedure (procedure date) was considered as a main confounder of the analyzed coherences. Additional PV angiography was performed as indicated by the operator with the intention to reduce angiographies where possible. Therefore, performed PV angiography was considered a possible confounder and a sub analysis assessed predictive value of performed PV angiography on the outcome parameters and PV angiography.

### Confirmatory statistical analysis

Initial approach to analyze impact of software version on (after transformation) normally distributed outcome parameters (procedure duration, fluoroscopy duration, dose area product) was analysis-of-variance (ANOVA). A Tobit regression model was fitted to assess the effect of software version on used contrast as a censored outcome variable. Additional sensitivity analysis was performed to evaluate the impact of the two possible confounders PV angiography and procedure date. For normally-transformed outcome parameters a general linear model (GLM) adjusting for procedure date and performed PV angiography was calculated. If both ANOVA and adjusted GLM were significant for effects of software version on the outcome, post-hoc testing was performed for ANOVA using Turkey-correction for multiple testing. Results were interpreted accordingly. Sensitivity analysis for the left-censored variable used volume of contrast die was analyzed via adjusting the initial Tobit-regression model (model 1: contrast_dye ∼ software_version) for both confounders (model 2: contrast_dye ∼ software_version + procedure date + PV_angiography) and calculating a third model with both confounders but without software version (model 3: contrast_dye ∼ procedure_date + PV_angiography). Model 2 and model 3 were compared in a Likelihood-Ratio-test to assess superiority of model 2.

**Supplementary Table 1:** Results of Tobit regression analysis with and without adjustment for possible confounding covariates. Reported are estimates with 95% confidence intervals and resulting adjusted p-values.

| **Model 1:** contrast_volume ~ software_version | | | |
| --- | --- | --- | --- |
|  | **Estimate** | **95% Confidence Interval** | **p-value** |
| v 1.4.8 | -34.9 | (-53.6, -20.7) | < 0.0001 |
| v 1.4.7 | -11.5 | (-26.8, 3.8) | 0.1403 |
| v 1.4.6a | -37.2 | (-48.9, 20.9) | < 0.0001 |
|  |  |  |  |
| **Model 2:** contrast_volume ~ software_version + epu_date + pv_angio | | | |
|  | **Estimate** | **95% Confidence Interval** | **p-value** |
| v 1.4.8 | -61.3 | (-115.1, -7.5) | 0.0263 |
| v 1.4.7 | -34.8 | (-67.9, -1.7) | 0.0402 |
| v 1.4.6a | -33.5 | (-54.8, -12.2) | 0.0022 |
| Procedure Date | 0.0 | (0.0, 0.0) | 0.0349 |
| PV angiography | 35.9 | (23.8, 48.0) | < 0.0001 |

**Supplementary Figure 1:** Post-hoc analysis results of ANCOVA analysis performed to evaluate the impact of different software versions on fluoroscopy duration. ANCOVA was adjusted for PV angiography and procedure date.


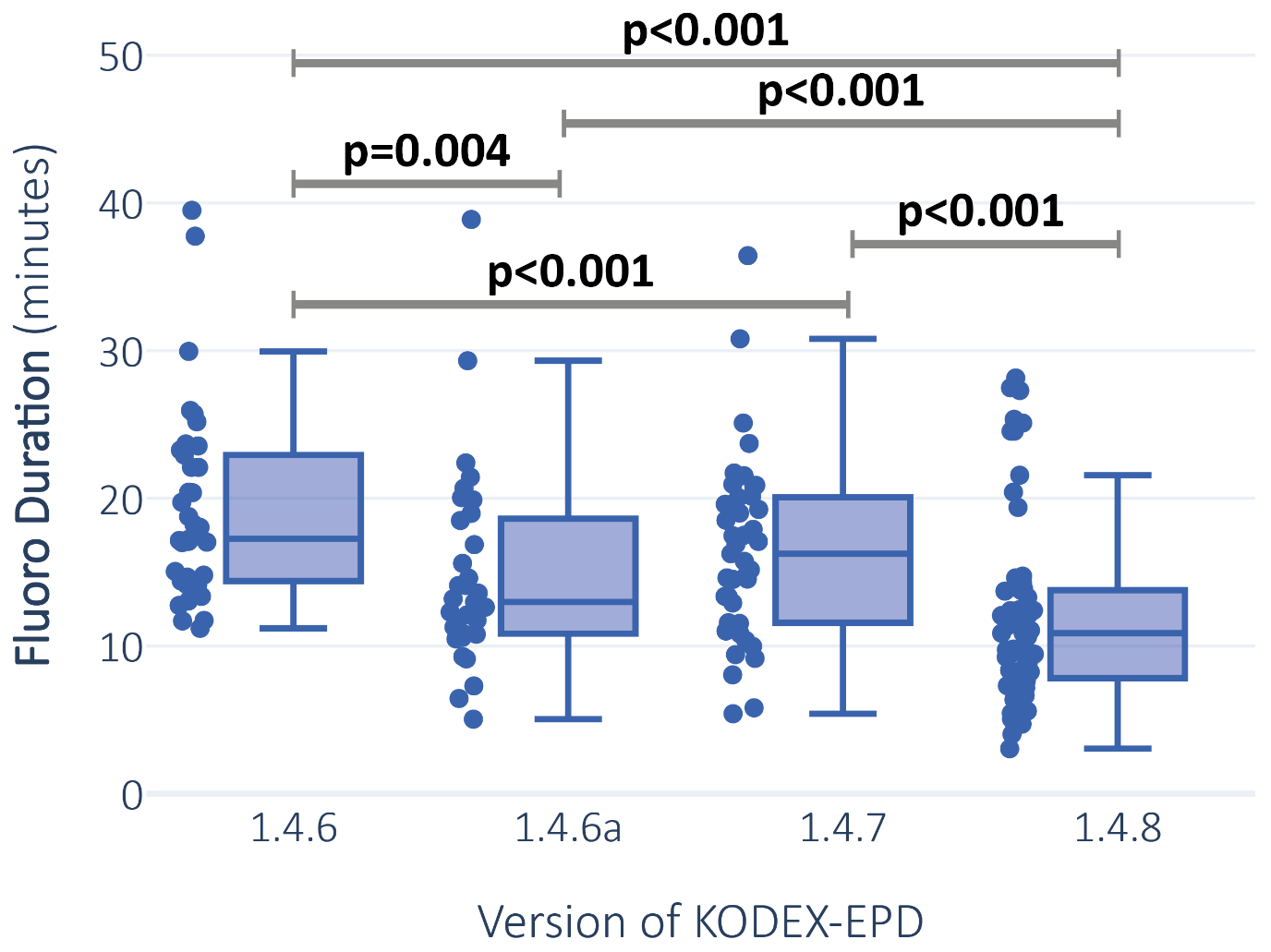

Supplement: Supplementary file 1 [file Data_Sheet_1.DOCX]
